# Changes in the Food-Related Behaviour of Italian Consumers during the COVID-19 Pandemic

**DOI:** 10.3390/foods10010169

**Published:** 2021-01-15

**Authors:** Rosa Maria Fanelli

**Affiliations:** Department of Economics, University of Molise, 86100 Campobasso, Italy; rfanelli@unimol.it

**Keywords:** COVID-19, daily lifestyle, eating habits, food consumption behaviour, Italian consumers

## Abstract

The principal aim of this study is to explore the effects of the first lockdown of the Coronavirus Disease 2019 (COVID-19) pandemic on changes in food consumption and food-related behaviour on a diverse sample of Italian consumers aged ≥18 years. To achieve this aim, the research path starts with an investigation of some of the first few studies conducted on Italian consumers. It then reports the findings of a pilot survey carried out on a small sample of Italian consumes who live in Molise. The studies chosen for investigation were published as articles or research reports. In total, six relevant studies were chosen, each involving a different sized sample of Italian consumers. The average number of respondents is 2142, with a standard deviation of 1260.56. A distinction is made between the results of the articles, the research reports, and the pilot survey. The latter was conducted to develop and validate the components of a new questionnaire and, furthermore, to assess changes in the eating habits of individuals during the COVID-19 pandemic. The results suggest that the effects of the pandemic on consumer behaviour can, above all, be grouped into changes related to shopping for food, eating habits, and food-related behaviour. This article can serve as the basis for future research in this area as it identifies and highlights key changes, in addition to comparing the earliest evidence available, using a critical approach.

## 1. Introduction

This paper analyses and makes a first evaluation of the consequences of the Coronavirus Disease 2019 (COVID-19) pandemic on the eating habits and food-related behaviour of different samples of Italian consumers. As is well-known, the outbreak of severe acute respiratory syndrome-coronavirus-2 (SARS-Cov2), or COVID-19, which was eventually declared a pandemic by the World Health Organization (WHO) [1], has impacted the daily life of many people. With the aim of limiting the spread of COVID-19, individuals quickly changed their lifestyles and, in particular, their eating habits. Indeed, in Italy, the instruction to stay at home and go out only if strictly necessary, imposed by the Decree “#Io resto a casa” [2] (translated as the #stayathome decree), had considerable implications for the eating habits of many people living in Italy, and saw a drastic reduction in any form of socialisation. The limitation of social interaction and, consequently, the self-isolation, changed the daily routines and, accordingly, the eating habits of many people.

Indeed, on the consumer side, as stated by the latest report by the Istituto di Servizi per il Mercato Agricolo-alimentare (Ismea) [3], during the period of confinement, the limited access to daily grocery shopping caused a reduction in the consumption of fresh foods, especially fruit, vegetables, and fish. At the same time, there was an immediate instinctive reaction to hoard food, with consumers tending to buy conservable products (e.g., pasta, rice, frozen foods, preserved tomatoes, and so on). In addition, during the initial phase of the crisis, there was a move towards fourth and fifth range products (vegetables and ready-made pizzas). Overall, in the weeks from 17 February to 15 March, Italians’ spending on packaged products exceeded that of the previous four weeks by 17% and that of the same weeks in 2019 by 19%; in terms of modern distribution, during this period of emergency, in which the Hotellerie, Restaurant, Catering (Ho.Re.Ca) channel was gradually shut down, about 750 million more euros were spent compared with the norm (similar period 2019). With regard to purchases, however, the increased orientation of many consumers towards online shopping (+57% in the penultimate week of February, +81% in the last of February, and +97% in the second week of March) brought about difficulties for the delivery system, which in turn led to increased purchases in large-scale distribution (GDO) and in neighbourhood shops [3]. Conversely, there was a reduction in purchases at super- and hypermarkets as they were considered too crowded and less safe than smaller stores. However, in terms of distribution formats, the greatest increase in sales was recorded in supermarkets (+23% from 17 February to 15 March on an annual basis), where almost half of purchases took place (43%), and in discount stores (+20%), where about a fifth of buyers went. Significant increases were also seen in small-scale stores, where sales increased overall by 17% compared with the previous year. The increasingly stringent limitations on travel favoured spending in nearby stores and, therefore, the growth of this channel was even more evident in the short-term (+20% compared with the previous month). The growth in turnover in hypermarkets was less intense; these stores were penalized in part by the closure of shopping centres within which many of them are located (+11% on an annual basis).

The same Ismea report highlights a substantial and progressive reduction in growth in local markets, many of which closed in the absence of fixed structures [3]. Furthermore, some studies present in the international literature suggest that the first wave of the COVID-19 pandemic had consequences for the functionality of healthcare; led to losses for national and international economic business; and impacted the provision of social services such as movie and play theatres, sports clubs, gymnasiums, and swimming pools. There were also effects for dietary change patterns, lifestyle behaviours, diet, eating, and physical activity [4,5,6,7,8]. Indeed, one research report [9] indicates that consumers were deeply concerned about the impact of COVID-19, from both a health and an economic perspective. However, they were more fearful of the economic impact than for their health.

Other authors have focussed more on the demand side and have highlighted how the COVID-19 pandemic progressively changed the way people purchased and consumed their food. Firstly, during the initial stage of the pandemic, consumers focused on panic buying to mitigate the risk of future shortages [10,11]. It is also possible, in accordance with Cranfield [12], that some consumers—wanting to reduce the number of shopping trips, and hence reduce their exposure to the virus and their risk of contracting it—stockpiled food, especially long-life milk, pasta, rice, and tinned vegetables [13]. Consequently, this exacerbated stockout situations and often led to price increases for consumer products [14]. Secondly, the first lockdown period caused changes in people’s consumer habits and in the quality of their diet due on the one hand to an orientation towards the consumption of processed food such as convenience foods, junk foods, snacks, and ready-to-eat cereals [15], and on the other, to a reduction in availability of some products such as fruits and vegetables [16]. Thirdly, the health emergency influenced the methods of purchasing food products. Many people perceived the physicality of shopping in a grocery store as a risk and rapidly shifted to online shopping [17].

Given the above scenario, the following research questions (RQs) were devised:

RQ_1_: How has COVID-19 determined changes in eating habits and food-related behaviour for Italian consumers?

RQ_2_: How can a short questionnaire with a sufficient number of items be designed able to assess changes in eating habits during the first lockdown period?

## 2. Materials and Methods

To answer the two RQs outlined in the introduction, this paper draws on some earlier surveys conducted in Italy during and after the first COVID-19 emergency period. These empirical studies consist of the most relevant pieces of research that investigate changes in eating habits and changes in food-related behaviour. The analysis is inspired by the necessity to compare, using a critical approach, the results of these first studies.

Once the research to be analysed was identified, an accurate review of these published studies was needed to formulate qualitative and quantitative questions, which then constituted the source for the construction of a new questionnaire to be utilized for the pilot survey. Therefore, a standardised methodology was adopted. It included two phases: a comparative analysis of the first studies conducted on Italian consumers, as well as a pilot survey. The investigators have known information about this experiment.

### 2.1. Phase 1: A Comparative Analysis of the First Studies Conducted on Italian Consumers

A recent and comprehensive literature review using Google was carried out to gain a wider picture of the existing evidence of the impact of COVID-19 on the eating habits and food-related behaviour of Italian consumers (RQ_1_). Only two key phrases, “*Changes in eating habits during COVID-19 period*” and “*Food-related behaviour during the COVID-19 period*”, were included in the search, which produced 100 related articles. Following further screening of the titles, abstracts, and full-texts, six articles and reports were found to be relevant for this type of analysis.

The 94 articles not considered had as their main objective an evaluation of the impact of COVID-19 on people’s physical and mental health and did not include all the keywords. Questions were identified from the relevant articles and 18 items were generated to be used to construct a new questionnaire (RQ_2_).

### 2.2. Phase 2: Validation of the Questionnaire

In this phase, the pilot survey was conducted to validate the questionnaire. The latter was administered in the Italian language to 110 individuals aged 18 years and above. A Google form was prepared and online data were collected from 1 June to 31 August 2020, after the first lockdown period. The survey included an introductory page describing the objectives of the research to inform the respondents. The inclusion criteria for the respondents were as follows: residence in Molise, aged 18–80 years, and female or male.

The structured questionnaire included 36 questions, divided into six different sections:

(1) Personal and general data (including eight questions: age, gender, information on province and municipality of residence, marital status, level of education, occupation, and type and work activity);
(2) How eating habits had changed (including five questions: Did your work performance change? How much did you eat during the lockdown period? Did you eat healthily? Did you cook? Did you always read food labels?);
(3) The contents of the shopping basket: What did you buy less or more of than before? (including seven questions related to different categories of food and products purchased);
(4) The location of purchases (including four questions to find out where food shopping was done);
(5) Price changes (including questions relating to eight principal foodstuffs to find out if there had been any price changes);
(6) Food waste (including four questions to find out if more or less food had been thrown away during the lockdown).

The questionnaire was anonymous.

### 2.3. Statistical Analysis

Data analysis was then carried out using the STATA programmer (Stata 12.32, software package created in 1985 by Stata Corp) in order to answer the study RQs. Descriptive statistics were then employed to explore the socio-demographic characteristics and changes in eating habits. The data are represented as numbers and percentages in parentheses (%). The results of the research are analysed, graphically represented, and interpreted in the following Section 3 and Section 4.

## 3. Principal Results of Phase 1

This section describes the results of the study in response to the RQs. As is well-known, many studies use questionnaires as a means of acquiring information. The quality of the survey results depends on the willingness of the participants to respond to the questions. In contrast, the level of comparison of the analysis results depends on the questions reported in the questionnaire.

For this study, the main surveys conducted on Italian consumers during and after the first lockdown period were considered (Table 1).

The data reported in Table 2 show how the various surveys involved a variable number of Italian consumers and revealed different information. The Consiglio per la Ricerca in Agricoltura e l’Analisi dell’Economia Agraria (CREA) survey focusses more on the demographic characteristics of consumers and on the different types of products consumed [18]. In contrast, the report prepared by the Istituto Zooprofilattico Sperimentale delle Venezie (IZSVe), gives more importance to the methods of purchasing products, the handling of food by consumers, the risks due to different diets on weight gain, and the importance attributed to food safety [19]. The comparison between the main information extrapolated from two articles by Di Renzo et al. and Scarmozzino and Visioli, on the other hand, highlighted that the former [20] is more in line with the CREA report, while the latter [21] had similar findings to the IZSVe report. Survey 3 [22] gives more importance to shopping for food. Finally, Survey 6 [23] concentrates on food as an antidote to anxiety. In more detail, the various investigations bring to light, albeit in different ways, how the period of confinement changed the eating habits and lifestyles of Italian consumers.

Beginning with the socio-demographic profile of the respondents, the information reported in Table 2 highlights that, in Surveys 2 and 3, respondents were represented mainly by females, by people living with their parents, by people with a high level of study, and by those with an average age equal to 46 years. In the other surveys, this information was not detected (n.d).

**Table 1:** The first relevant surveys conducted on Italian consumers. COVID-19, Coronavirus Disease 2019.

N.	Surveys	References	
1	Istituto Zooprofilattico Sperimentale delle Venezie	IZSVe, 2020	[19]
2	Consiglio per la ricerca in agricoltura e l’analisi dell’economia agraria	CREA, 2020	[18]
3	Effetto LOCKDOWN: Come sono cambiate le abitudini alimentari degli italiani durante l’emergenza COVID-19	Fontefrancesco M.F. et al., 2020	[22]
4	COVID-19 and the Subsequent Lockdown Modified Dietary Habits of Almost Half the Population in an Italian Sample	Scarmozzino F. and Visioli F., 2020	[21]
5	Eating habits and lifestyle changes during COVID-19 lockdown: an Italian survey	Di Renzo L. et al., 2020	[20]
6	Psychological Aspects and Eating Habits during COVID-19 Home Confinement: Results of Eating Habits and Lifestyle Changes- COVID-19 (EHLC-COVID-19) Italian Online Survey	Di Renzo L. et al., 2020	[23]

**Table 2:** Socio-demographic profile of respondents, absolute value (N.) and (%) on the total.

Survey	1	2	3	4	5	6
N. Participants	730	2900	3155	1932	3533	602
Female	n.d.	2175 (75)	2135.85 (67.7)	n.d.	n.d.	n.d.
Male	n.d.	725 (25)	1041.15 (33)	n.d.	n.d.	n.d.
Lives with parents	n.d.	2465 (85)	n.d.	n.d.	n.d.	n.d.
Lives alone	n.d.	319 (11)	n.d.	n.d.	n.d.	n.d.
30–49 years	n.d.	1119.4 (38.6)	n.d.	n.d.	n.d.	n.d.
50–69 years	n.d.	1044 (36)	n.d.	n.d.	n.d.	n.d.
18–29 years	n.d.	696 (24)	n.d.	n.d.	n.d.	n.d.
Average age	n.d.	n.d.	46	n.d.	n.d.	n.d.
University degree	n.d.	1972 (68)	n.d.	n.d.	n.d.	n.d.
High school diploma	n.d.	826.5 (28.5)	n.d.	n.d.	n.d.	n.d.

Source: own processing of previous studies, information not detected is indicated with (n.d.).

Regarding food shopping (Table 3), while Surveys 1 and 3 investigate mainly where the participants bought their food, in the other four surveys, this information was not detected. In particular, Survey 1 found that a considerable number of participants (175) bought their food online and about 43% (311.71 participants) had decreased their purchase of ready-to-eat foods. In contrast, the main findings of Survey 3 were that, during the first lockdown period, 44% (1388.2) of participants had favoured small retail stores for their food shopping, had increased their frequency of food shopping, and had done more food shopping online. Another interesting piece of information reported by Survey 5 was that consumers bought fresh products, mainly fruit and vegetables, from organic farms. Survey 6 had omitted this type of information.

Table 4 shows how the contents of shopping baskets changed during the quarantine period. Surveys 2 and 3 noted, on the one hand, an increase in the excessive consumption of unhealthy foods like sweets and salty snacks, but, on the other hand, there was also an increase in the consumption of healthy foods such as fresh fruit and vegetables.

Another important finding concerns alcohol consumption. While Surveys 1 and 3 registered a decrease, Surveys 2 and 4 registered an increase. A decrease in alcohol consumption could be an indicator of physical and subjective well-being [24]. Counter to this, an increase could be due to the fact that alcohol helps fight boredom and anxiety resulting from the measures introduced by the government to minimize the spread of the virus and, in accordance with Fairbairn and Sayette [25], by interpersonal isolation.

Confinement and stress have also led individuals to consume unhealthy foods to a greater extent, which was a finding of Surveys 4, 5, and 6 (Table 5). This is supported by previous studies that have highlighted how stress leads to overeating, especially the consumption of comfort foods rich in sugar, leading to food cravings [26,27,28]. Quarantine induces anxiety and stress [29]. Furthermore, consumers often manifest impulsive behaviour, purchasing products that they consider absolutely necessary [30].

During the lockdown, Italians had a greater desire to cook and bake as an antidote to boredom. Indeed, home cooking was rediscovered and the time spent preparing food increased, as shown mainly by the findings of Surveys 1 and 2 (Table 6).

Regarding changes in diet, Surveys 1 and 2 found that, for 14% and 31% of respondents, respectively, diets changed. In contrast, however, Survey 1 found that 50% (365) of respondents had not modified their diet during the lockdown (Table 7).

Maintaining good nutrition is crucial, above all, in a period in which the immune system might need to fight off infection. In particular, Survey 5 found that, for this reason, during the lockdown period, the majority of participants (63%) had adhered strictly to the Mediterranean diet, and 15% (540) followed a healthier diet (Table 8). In addition, Italians favoured Mediterranean foods, and the nutritional quality of diets remained high. In accordance with [31], the need to consume healthier food emerged as an important concern during the first phase of the pandemic. It should be remembered that a key health benefit of the Mediterranean diet can be attributed to the now-disappearing habit [32] of preparing most meals at home and sharing them with friends and family, thereby contributing to a pleasant social environment.

The lifestyles of many Italians substantially changed owing to the containment measures, and there was also a risk of increased sedentary behaviour. The results of four surveys found, in accordance with Panahi and Tremblay (2018) [33], that low physical activity levels can interact with both body fat and appetite dysregulation (Table 9). During the lockdown, the closure of gyms and fitness centres and restrictions imposed on visiting parks, playgrounds, and so on to curb the spread of COVID-19 limited access to many forms of physical activity.

During the lockdown period, as highlighted by the findings of Survey 4, about 43% of the participants in this survey declared that they had great difficulty finding certain products (such as fresh products) and 6.4% encountered price increases for these same products (Table 10). This may have been due to the enormous difficulties for the agricultural supply chain in procuring fresh products, mainly fruit and vegetables, and to the fact that the agro-food market is dependent on imports. In addition, the manifestation of some critical situations can destabilize the food supply of the population. This meant that, during the quarantine, there was a reduction in the consumption of fresh food.

Only Surveys 1 and 4 investigated food safety. In the former, 70% of respondents declared that food safety was an important attribute of food products, especially during a health emergency; in the latter, only 1.3% were of the same opinion. Thirty percent of participants in the first survey agreed that food safety was not that important (Table 11).

## 4. Novel Results of Phase 2

### 4.1. Socio-Demographic Profile of Respondents

A total of 110 Italian consumers participated in the pilot survey. The questionnaire was completed in its entirety by 50 of the respondents (45.5% of the total). The questions without answers tended to be those relating to changes in the contents of the shopping basket and changes to food shopping. On the basis of the answers given in the first section of the questionnaire, respondents were from the two provinces of Molise: Campobasso (92%) and Isernia (8%). The sample was gender unbalanced (70% female and 30% male). This could perhaps be attributed to the prevalence of the idea during the first lockdown that daily grocery shopping was a woman’s responsibility. Table 12 shows a summary of the demographic characteristics of the participants. Participants ranged in age from 18 to 80, with an average age of 47 years. By comparison, the median age for the Italian population is approximately 44.91 [34].

Overall, the sample is highly educated, as 48% and 36% of the respondents possessed a high school diploma and university degree, respectively. In contrast, only 14% had a primary school diploma, and 2% had no qualifications.

The majority of respondents (58%) lived with partners, 30% were from single person households, 8% were divorced, and 4% were widowed. As for household composition, those that were made up of 3–4 people represented 64% of the total sample, 28% of households were made up of 1–2 people, while 8% were composed of 5–6 people, and none had more than seven members. This indicates that medium-sized and large families still dominate the regions of central Italy.

Territorial coverage spreads over all municipalities of Molise. Here, 64% of respondents live in municipalities with a population of between 5001 and 10,000 inhabitants, 34% in towns with up to 5000 inhabitants, and only 2% between 10,001 and 50,000.

In terms of the employment status, of all the people interviewed, 74% were employed, 12% were self-employed, 6% were students, 4% were homemakers, and 4% were pensioners. Eighty-six percent of respondents said they had a full-time job. The majority of respondents (52%) had a sedentary lifestyle. In general, 32% of those surveyed said they had suspended their work activities during the quarantine, nothing had changed for 28%, 20% had moved exclusively to home-working, 18% had worked in a mixed mode, and finally only 2% said they had lost their jobs.

### 4.2. Changes in Eating Habits and in Food-Related Behaviour

Regarding eating habits, 60% (30) of the surveyed individuals declared that the quarantine had not changed the amount of food they consumed, while 34% reported eating more, and 6% said they had eaten much more. The majority of the respondents (60%) continued to eat healthily and 20% and 4% said they had eaten more healthily and much more healthily than before quarantine, respectively.

**Figure 1:**
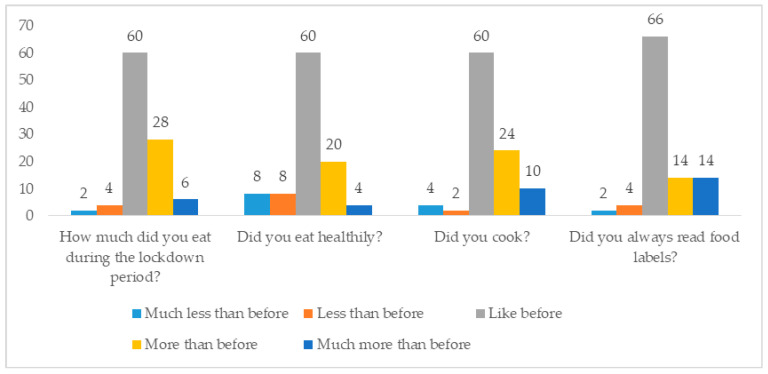
Changes in eating habits (%). Source: own processing of pilot survey.

During confinement, the desire to cook increased more and much more than before for 34% of participants. Finally, 28% of respondent read food labels more or much more than before (Figure 1).

**Figure 2:**
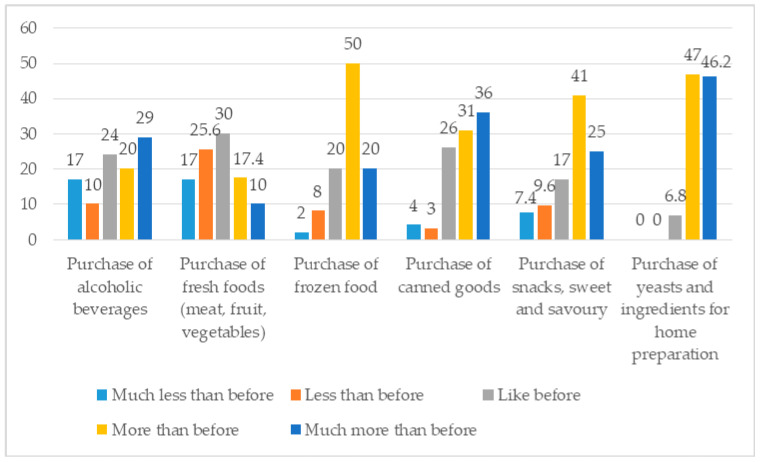
Changes in food purchase (%). Source: own processing of pilot survey.

As presented in Figure 2, during confinement, a considerable number of participants indicated that they purchased the following foods more, and much more frequently than before: frozen food (70%); ingredients for home preparation (93.2%); canned foods (67%); sweet and savoury snacks (65%); and alcoholic drinks (49%).

**Figure 3:**
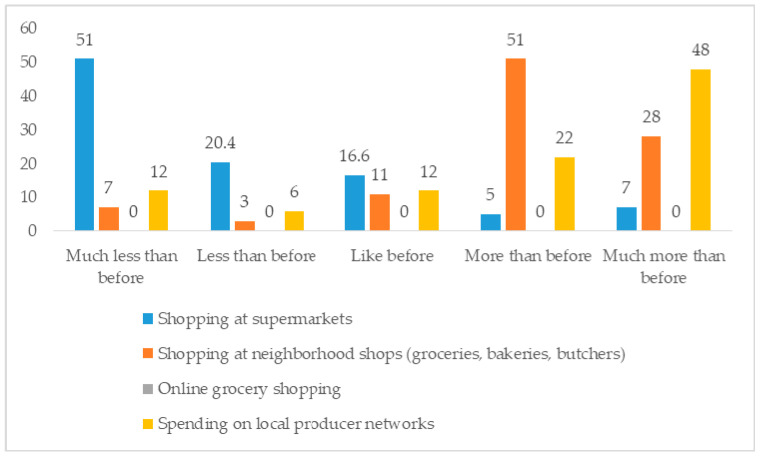
Changes in food shopping (%). Source: own processing of pilot survey.

In relation to the choice of distribution channels, after an initial, almost exclusive orientation towards large-scale distribution, there was a subsequent passage to neighbourhood shops (including fruit and butcher shops), both because of the introduction of restrictions on movement and because they often had shorter queues. These shops were also sometimes considered safer than larger, busier environments. Hypermarkets were particularly penalized in the months of lockdown because they were located within shopping centres in which all other stores had been closed, further discouraging consumers from going there (Figure 3).

**Figure 4:**
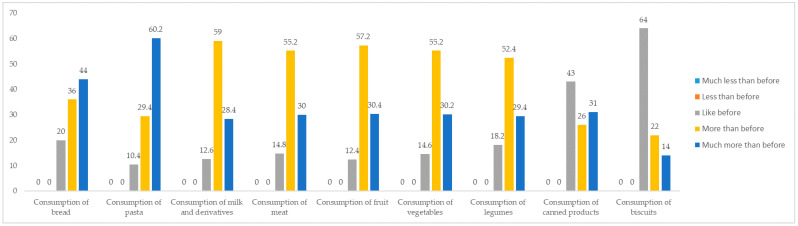
Changes in food consumption. Source: own processing of pilot survey.

As shown in Figure 4, the COVID-19 pandemic had effects for the food consumption of the respondents. By analysing the figures for the more specific semantic tags, it is possible to notice that, during the quarantine period, about 90% of the respondents considerably increased (including more and much more) their consumption of pasta and fruit; 87.4% increased their consumption of milk and derivatives; and 85.4% increased their consumption of vegetables. Furthermore, for all other categories of food product considered, participants indicated that their consumption had reduced. This suggests that consumers became more health conscious.

**Figure 5:**
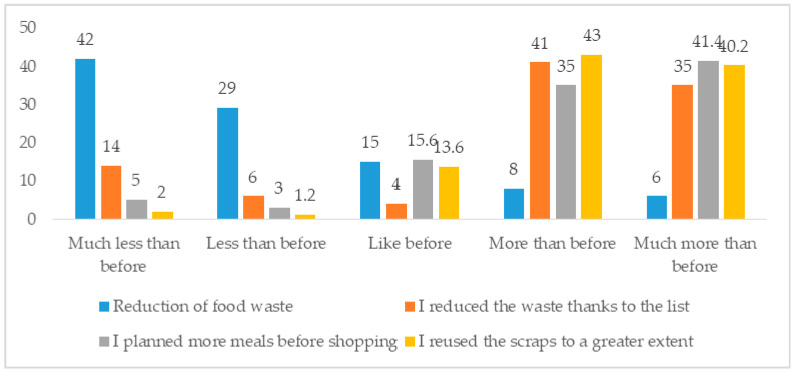
Changes in attention given to food waste (%). Source: own processing on pilot survey.

Finally, growing concern for the general economic conditions produced what can be termed an “accountability” in consumer behaviour, which led to a sharp reduction in the amount of food wasted (Figure 5).

## 5. Discussion and Conclusions

This study provides novel information about changes in eating habits and food-related behaviour in Italy during the first lockdown period of the COVID-19 pandemic.

To the best of my knowledge, the analysis is among the first to compare the results of different surveys conducted in Italy to investigate the immediate impact of the COVID-19 lockdown on the eating habits and food-related behaviour of Italian residents. Indeed, this paper presents an analysis of the initial impact of COVID-19 on the food consumption of different samples of Italian consumers. It should be noted that the reliability of the results presented in the different surveys considered in the analysis is influenced by the attention paid to the different aspects of Italian consumption (socio-demographic consumer profile, purchases of food products, changes in the composition of the shopping basket, the use of food as an antidote to anxiety, the desire to cook and bake, the need to maintain good nutrition, changes in physical activity, and attention given to food safety).

To begin with the socio-demographic characteristics of the respondents, they were mostly female, were living with their parents, had a high level of education, and had an average age equal to 46 years (Section 3). This is in line with the novel findings of the pilot survey (Section 4).

Regarding eating habits and food-related behaviour, during the lockdown period, most participants in the pilot study indicated that they had not changed their diet. However, the surveys noted changes in the purchase of food products. With people spending more time at home and dining out becoming less accessible, it is possible to notice a major shift in people’s attitudes and behaviour concerning food and health. Indeed, there have been clear changes in the way consumers eat, shop, and interact with food.

**Figure 6:**
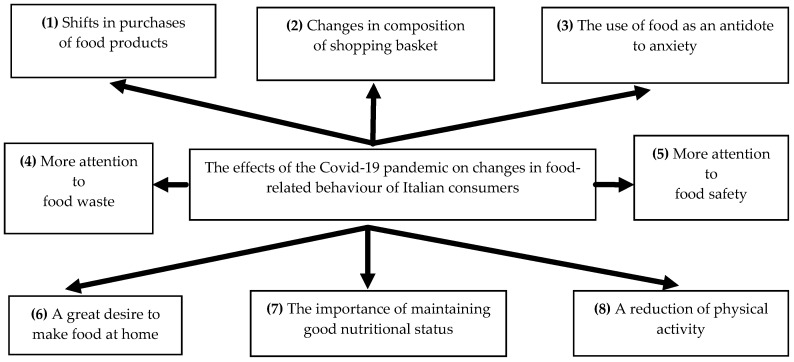
The effects of the COVID-19 pandemic on changes in eating habits and lifestyle.

The research highlighted several key consumer trends that are currently shaping food and health habits in Italy (Figure 6).

(1) First, the results indicate a shift towards the purchase of food in small retail stores and an increased frequency of food shopping during the COVID-19 pandemic. The results of the pilot study indicate that this is likely because of the fact that, during the pandemic, super and hypermarkets were considered to be too crowded and less safe than smaller stores. This is supported by the findings of previous surveys.
(2) Consumers reduced their purchases and consumption of ready-to-eat foods. At the same time, they adhered strictly to the Mediterranean diet and ate more healthily, including in their diets more fresh fruit and vegetables. The findings are in line with those of the pilot survey and are in contrast to those highlighted by [3].
(3) The results indicate that there is, in accordance with other studies in the international literature [35,36,37,38], a relationship between stress and emotional eating. It has been shown that people under stress crave more high fat and high sugar foods, as a stressed body needs more energy to function. Indeed, the results of the pilot survey are in line with those of previous studies; the COVID-19 economic crisis is affecting diets, primarily through declining demand for vegetables, fruits, and animal-sourced foods, which are the main sources of essential micronutrients in diets.
(4) The attention given to food waste is an important aspect of food consumption, especially in a period of economic restrictions [39,40]. Indeed, the findings of the pilot survey highlight what can be termed an “accountability” in consumption, which led to a sharp reduction in the amount of food wasted. This aspect of the pandemic was completely absent from the principal surveys conducted on Italian consumers.
(5) Another important issue considered by previous surveys, but not detected in the pilot survey, is the attention of consumers to food safety. Many participants (70%) in Survey 1 declared that food safety was an important attribute of food products, especially during a health emergency. The findings of the surveys analysed highlighted great support for food safety, with consumers aiming to get the freshest possible food for their families.
(6) The results of this study indicate an increase in home cooking during the COVID-19 pandemic. Participants started preparing more food at home (or their wives and mothers did), resulting in a reduced reliance on a housekeeper for cooking purposes. These results match those observed by the pilot study (that found about half of the participants reported they were cooking and baking more during the pandemic). It seems possible that this rise in home cooking is related to attempts to occupy the increased free time resulting from quarantine.
(7) Good nutrition is always important, but during this pandemic, it has been given even more importance because a well-balanced diet of nutritious food helps support a strong immune system [38]. It is still possible to purchase and consume healthy food during these difficult times.
(8) The closure of gyms and fitness centres and restrictions imposed on visiting parks, playgrounds, and so on to curb the spread of COVID-19 limited the access to many forms of physical activity.

With all these relevant changes on the food front for consumers during the first wave of the pandemic, the pressing question becomes which of these trends will stick or even accelerate, especially given what may prove an extended period of intermittent social distancing. For the younger digital generations, relying on the large footprints of supermarkets may become a thing of the past. Furthermore, the lockdown seems to have influenced people’s ability to control their relationship with food.

In addition, the results of the pilot survey conducted on a restricted sample of Italian consumers during the pandemic indicate that COVID-19 and the subsequent lockdown induced a third of respondents to eat more, or much more, than before the quarantine. Indeed, the lockdown caused by the COVID-19 pandemic heavily influenced our lives by completely changing our routines and isolating us from our loved ones. It is now important that the government considers the need for nutritional education, programmes, and campaigns, particularly during this second critical period of the pandemic in Italy.

This study has some strengths and limitations that should be acknowledged. The first strength is that it provides a comparative analysis of the most important surveys conducted on different samples of Italian consumer and offers an interesting reflection on a possible, larger future survey that might be carried out following the second period of lockdown. The second strength is that the pilot survey, conducted quickly after the first critical period of the lockdown in Italy, tested a structured questionnaire with questions that were absent in previous surveys, giving novel results concerning a sample of consumers living in Molise.

There are also limitations. One is the median age (46 years) of the participants in the surveys considered, which is probably because of the fact that the questionnaire was distributed through social media, and thus was aided by the respondents’ familiarity with digital technologies. Another limitation is that the sample of the pilot survey is very restricted as many of the respondents did not complete the questionnaire in its entirety. Finally, as the COVID-19 pandemic is still ongoing, the data need to be confirmed and investigated in the future with larger population studies.

To conclude, this article reports, using a critical approach, the first evidence of changes in eating habits and food-related behaviour and can serve as a basis for future research on the topic. Indeed, further studies are required to better understand the mechanisms behind these changes.

## Figures and Tables

**Table 3:** Shopping for food, absolute value (N.) and (%) on the total.

Survey	1	2	3	4	5	6
N. Participants	730	2900	3155	1932	3533	602
Shopping for food in small retail stores	n.d.	n.d.	1388.2 (44)	n.d.	n.d.	n.d.
Shopping for food online	175.2 (24)	n.d.	599.45 (19)	n.d.	n.d.	n.d.
Turned to organic farmers to purchase fruits and vegetables	n.d.	n.d.	n.d.	n.d.	529.95 (15)	n.d.
Increased frequency of food shopping	197.83 (27.1)	n.d.	851.85 (27)	n.d.	n.d.	n.d.
Increased quantities purchased	197.84 (27.1)	n.d.	n.d.	n.d.	n.d.	n.d.
Increased purchase of bread products	292 (40)	n.d.	n.d.	n.d.	n.d.	n.d.
Purchases of ready-made meals	n.d.	n.d.	n.d.	966 (50)	n.d.	n.d.
Decrease in the purchase of raw fish	n.d.	464 (16)	n.d.	n.d.	n.d.	n.d.
Decreased purchase of ready-to-eat foods	311.71 (42.7)	n.d.	n.d.	n.d.	n.d.	n.d.

Source: own processing of previous studies, information not detected is indicated with (n.d.).

**Table 4:** Changes in the shopping basket, absolute value (N.) and (%) on the total.

Survey	1	2	3	4	5	6
N. Participants	730	2900	3155	1932	3533	602
Low consumption of bread	n.d.	957 (33)	n.d.	n.d.	n.d.	n.d.
Excessive use of red meat (1–2 times a week)	n.d.	1856 (64)	n.d.	n.d.	n.d.	n.d.
Increased consumption of sweets (2 or more times a week)	n.d.	1290.5 (41.7)	n.d.	966 (50)	n.d.	n.d.
Increased consumption of salty snacks	n.d.	1696.5 (58.5)	n.d.	821.1 (42.5)	n.d.	n.d.
Increased consumption of fresh fruit and vegetables	n.d.	1798 (62)	n.d.	454.02 (23.5)	n.d.	n.d.
Increased consumption of pulses	n.d.	768.5 (26.5)	n.d.	n.d.	n.d.	n.d.
Increased consumption of water	n.d.	638 (22)	n.d.	n.d.	n.d.	n.d.
Increased consumption of olive oil	n.d.	623.5 (21.5)	n.d.	n.d.	n.d.	n.d.
Little consumption of pasta and brown rice	n.d.	n.d.	n.d.	409.58 (21.2)	n.d.	n.d.
Decrease in alcohol consumption	308.06 (42.2)	n.d.	252.4	n.d.	n.d.	n.d.
Increase in alcohol consumption	n.d.	464 (16)	n.d.	710.98 (36.8)	n.d.	n.d.

Source: own processing of previous studies, information not detected is indicated with (n.d.).

**Table 5:** Food as an antidote to anxiety, absolute value (N.) and (%) on the total.

Survey	1	2	3	4	5	6
N. Participants	730	2900	3155	1932	3533	602
Use of food to respond to anxious feelings	n.d.	n.d.	n.d.	195.13 (10.1)	n.d.	n.d.
Need to increase food intake to feel better	n.d.	n.d.	n.d.	n.d.	n.d.	293.17 (48.7)
Anxious feelings due to current eating habits	n.d.	n.d.	n.d.	n.d.	n.d.	331.7 (55.1)
Exclusion of foods that lead to anxious feelings	n.d.	n.d.	n.d.	n.d.	n.d.	347.96 (57.8)
Increase in stress/anxiety/boredom during quarantine	n.d.	n.d.	n.d.	n.d.	1215.35 (34.4)	n.d.

Source: own processing of previous studies, information not detected is indicated with (n.d.).

**Table 6:** Time spent preparing food, absolute value (N.) and (%) on the total.

Survey	1	2	3	4	5	6
N. Participants	730	2900	3155	1932	3533	602
Products are washed more (especially vegetables)	n.d.	n.d.	n.d.	n.d.	n.d.	122.21 (20.3)
More time is spent preparing food	127.75 (17.5)	1160 (40)	n.d.	n.d.	n.d.	n.d.
Home made products (bread, pasta, pizza and desserts)	118.26 (16.2)	899 (3.1)	n.d.	n.d.	n.d.	n.d.

Source: own processing of previous studies, information not detected is indicated with (n.d.).

**Table 7:** Changes in diet, absolute value (N.) and (%) on the total.

Survey	1	2	3	4	5	6
N. Participants	730	2900	3155	1932	3533	602
The diet changed	102.2 (14)	899 (31)	n.d.	n.d.	n.d.	n.d.
The diet did not change	365 (50)	n.d.	n.d.	n.d.	n.d.	n.d.

Source: own processing of previous studies, information not detected is indicated with (n.d.).

**Table 8:** Adherence to the Mediterranean diet, absolute value (N.) and (%) on the total.

Survey	1	2	3	4	5	6
N. Participants	730	2900	3155	1932	3533	602
Having a lower adherence to the Mediterranean diet	365 (50)	n.d.	n.d.	958.27 (49.6)	n.d.	n.d.
Having a medium adherence to the Mediterranean diet	n.d.	1740 (60)	n.d.	n.d.	765 (21.7)	n.d.
Having a higher adherence to the Mediterranean diet	n.d.	n.d.	n.d.	n.d.	2228 (63.1)	n.d.
Healthier diet	n.d.	n.d.	n.d.	n.d.	540 (15.3)	n.d.

Source: own processing of previous studies, information not detected is indicated with (n.d.).

**Table 9:** Changes in activities and in eating habits, absolute value (N.) and (%) on the total.

Survey	1	2	3	4	5	6
N. Participants	730	2900	3155	1932	3533	602
Health risks due to reduced physical activity	365 (50)	n.d.	n.d.	n.d.	n.d.	n.d.
The perception of weight gain	511 (70)	1537 (53)	n.d.	n.d.	n.d.	n.d.
Eating more during quarantine	405.88 (55.6)	1276 (44)	n.d.	376.74 (19.5)	1717.04 (48.6)	n.d.
Not eating more during quarantine	n.d.	n.d.	n.d.	1022.03 (52.9)	n.d.	n.d.
Less appetite	n.d.	n.d.	n.d.	909.97 (47.1)	n.d.	n.d.
More appetite	n.d.	n.d.	n.d.	n.d.	627	n.d.

Source: own processing of previous studies, information not detected is indicated with (n.d.).

**Table 10:** Difficulty to find certain products, absolute value (N.) and (%) on the total.

Survey	1	2	3	4	5	6
N. Participants	730	2900	3155	1932	3533	602
A great difficult to find certain products	n.d.	n.d.	n.d.	824.96 (42.6)	n.d.	301 (50)
The increased price of certain products	n.d.	n.d.	n.d.	123.65 (6.4)	n.d.	n.d.

Source: own processing of previous studies, information not detected is indicated with (n.d.).

**Table 11:** Food safety, absolute value (N.) and (%) on the total.

Survey	1	2	3	4	5	6
N. Participants	730	2900	3155	1932	3533	602
Importance of food safety	511 (70)	n.d.	n.d.	25.12 (1.3)	n.d.	n.d.
Food safety is not important	219 (30)	n.d.	n.d.	n.d.	n.d.	n.d.

Source: own processing of previous studies, information not detected is indicated with (n.d.).

**Table 12:** Demographic breakdown of surveyed participants (*n* = 50).

**Age Classes (Years)**	
18–24, *n* (%)	3 (6)
25–31, *n* (%)	6 (12)
32–38, *n* (%)	6 (12)
39–45, *n* (%)	4 (8)
46–52, *n* (%)	7 (14)
53–59, *n* (%)	17 (34)
60–66, *n* (%)	6 (12)
67–73, *n* (%)	0 (0)
74–80, *n* (%)	1 (2)
Gender Female, *n (%*)/Male, *n* (%)	35 (70)/15 (30)
Province Campobasso, *n* (%)/Isernia, *n* (%) 46 (92)/4 (8)	
**Municipality of Residence (*n*. of Inhabitants)**	
until 5000, *n* (%)	17 (34)
5001–10,000 *n* (%)	32 (64)
10,001–50,000 *n* (%)	1 (2)
50,001–100,000 *n* (%)	0 (0)
100,001–1,000,000 *n* (%)	0 (0)
>1,000,000 *n* (%)	0 (0)
**Marital Status**	
Spouse/Cohabitant, *n* (%)	29 (58)
Single *n* (%)	15 (30)
Widower/Widow, *n* (%)	2 (4)
Separated or divorced, *n* (%)	4 (8)
**Educational Qualification**	
No title, *n* (%)	1 (2)
Elementary or high school diploma, *n* (%)	7 (14)
High school diploma, *n* (%)	27 (54)
University degree or higher qualification, *n* (%)	15 (30)
**Household (*n*. People)**	
0, *n* (%)	10 (20)
2, *n* (%)	4 (8)
3, *n* (%)	10 (20)
4, *n* (%)	22 (44)
5, *n* (%)	3 (6)
6, *n* (%)	1 (2)
>6, *n* (%)	0 (0)
**Profession**	
Employee, *n* (%)	37 (74)
Freelance, *n* (%)	6 (12)
Unemployed, *n* (%)	0 (0)
Retired, *n* (%)	2 (4)
Housewife, *n* (%)	3 (6)

Source: own processing of pilot survey.
